# Intervention with Polyvalent Bacterial Lysate Modulates T Helper Cell Subsets in Polish Children with Grass Pollen-Induced Allergic Rhinitis

**DOI:** 10.3390/biomedicines14071623

**Published:** 2026-07-19

**Authors:** Kamil Janeczek, Wioleta Grzegorzewska, Michał Zarobkiewicz, Dorota Suszczyk, Marek Mikołajczyk, Ewa Markut-Miotła, Izabela Morawska-Michalska, Adrian Bakiera, Aleksandra Chmielewska, Andrzej Emeryk, Jacek Roliński, Marta Rachel, Krystyna Piotrowska-Weryszko

**Affiliations:** 1 Department of Allergology and Pediatrics, Medical University of Lublin, 20-093 Lublin, Poland; 2 Department of Clinical Immunology, Medical University of Lublin, 20-093 Lublin, Poland; wioleta.grzegorzewska@umlub.edu.pl (W.G.);; 3 Independent Laboratory of Cancer Diagnostics and Immunology, Medical University of Lublin, 20-093 Lublin, Poland; 4 Department of Allergology, Voivodeship Rehabilitation Hospital for Children in Ameryka, 11-015 Olsztynek, Poland; 5 Department of Pharmaceutical Microbiology, Medical University of Lublin, 20-093 Lublin, Poland; 6 Independent Public Healthcare Centre of the Ministry of Internal Affairs and Administration in Lublin, 20-331 Lublin, Poland; 7 Department of Pediatrics, Lung Diseases and Rheumatology, University Children’s Hospital, 20-093 Lublin, Poland; 8 Institute of Medical Sciences, Medical College, Rzeszow University, 35-959 Rzeszow, Poland; 9 Department of Allergology and Cystic Fibrosis, State Hospital 2 in Rzeszow, 35-301 Rzeszow, Poland; 10 Department of Botany and Plant Physiology, University of Life Sciences in Lublin, 20-950 Lublin, Poland

**Keywords:** allergic rhinitis, grass pollen allergy, children, bacterial lysate, PMBL, T helper cells, immunomodulation

## Abstract

**Background:** Allergic rhinitis (AR) is a chronic condition that affects children’s quality of life. Studies show that adding bacterial lysates (BLs) to treatment can improve outcomes, but their effects on the immune system are still not fully understood. **Objectives:** This study aimed to evaluate the immunomodulatory impact of sublingually administered polyvalent mechanical BL (PMBL) on the expression of T helper (Th) cell-associated transcription factors and cytokines in children with grass pollen-induced AR. **Methods:** Immunological analyses were performed on blood samples collected during a previously conducted randomised, double-blind, placebo-controlled clinical trial (NCT04802616). Children aged 5–17 years with grass pollen-induced AR received either sublingual PMBL or placebo in three 10-day treatment cycles, each followed by a 20-day break. Peripheral blood mononuclear cells were collected at baseline before the grass pollen season (V1) and after completion of the study treatment during natural peak pollen exposure (V2) and analysed by flow cytometry to assess the expression of transcription factors and cytokines associated with Th1, Th2, Th10, Th17, and Treg-like immune responses. **Results:** In the PMBL group, there was a significant increase in the expression of T-bet, E4BP4, FoxP3, IFN-γ, and IL-10, along with a reduction in GATA3 and IL-4 expression (*p* < 0.001). The placebo group exhibited increased expression of GATA3 (*p* < 0.001), RORγT (*p* < 0.001), and IL-4 (*p* = 0.004) and a reduction in T-bet expression (*p* = 0.007). Between-group comparisons at V2 revealed significantly higher expression of Th1-, Th10-, and Treg-associated markers, and lower Th2- and Th17-associated markers in the PMBL group compared to placebo. **Conclusions:** Sublingual PMBL administration modulates the expression of Th cell-associated transcription factors and cytokines in children with grass pollen-induced AR, consistent with enhanced Th1-, Th10-, and Treg-like immune responses and reduced Th2-associated activity.

## 1. Introduction

Allergic rhinitis (AR) is defined by IgE-mediated hypersensitivity reactions of the nasal mucosa, which occur in response to specific airborne allergens such as pollen, dust mites, fungal spores, and animal dander [1]. It is a widespread condition that affects up to 40% of children worldwide [2,3]. The prevalence of pollen-induced AR increases with age during childhood, and this increase stabilises after 16 years of age. The disease is diagnosed in approximately 2% of children at the age of 4 years, and in subsequent years, nearly 20% of children are affected. Remission of the disease in childhood is uncommon. The prevalence of persistent pollen-induced AR at age 4–24 years is 74%, which means that once AR is diagnosed, the risk of persistent disease is high [4].

Clinically, AR manifests as symptoms such as nasal congestion, rhinorrhoea, sneezing, and nasal itch [1]. Although these manifestations seem trivial, they significantly impact children’s quality of life, productivity, and overall well-being [5]. A recently published meta-analysis demonstrated that children with AR have significantly shorter sleep duration and that sleep disturbances may co-occur with mental health problems [6]. In addition, AR often coexists with other allergic conditions such as asthma and eczema, creating a more complex clinical picture. Approximately 40% of patients with AR have a diagnosis of asthma; in these patients, poor control of nasal symptoms is a significant risk factor for asthma exacerbations. AR is associated with high health care costs, predominantly in terms of indirect costs related to reduced quality of life. It is estimated that in the United States alone, these costs amount to 5 billion dollars annually [7]. Therefore, early recognition, optimal interventions, and regular monitoring are pivotal in mitigating the symptoms of AR, reducing the risk of comorbidities, and improving overall quality of life. Children with AR require a comprehensive approach involving allergen avoidance, pharmacotherapy, and allergen immunotherapy. Currently, antihistamines, glucocorticoids, leukotriene receptor antagonists, and allergy vaccines are used [8].

Given the limitations of current therapies and the chronic nature of AR, new adjunctive approaches are being explored, including immunomodulatory agents such as bacterial lysates (BLs). They are classified as postbiotics and consist of antigens derived from inactivated bacteria that are the most common pathogens of the respiratory tract. For over 70 years, BLs have been successfully used in the prevention of recurrent respiratory tract infections in both children and adults, leading to a reduction in the number of acute respiratory infections, shorter duration of illness, and fewer courses of antibiotic therapy. Today, BLs are commonly used in clinical practice in many countries, particularly in Europe, as an adjunctive strategy for the prevention of recurrent respiratory tract infections [9,10]. Recent studies indicate that BLs may also be an additional therapeutic option for patients with AR. The addition of BLs to standard AR therapy reduces the severity of nasal symptoms and decreases the need for oral antihistamines and intranasal glucocorticoids [11,12,13,14,15,16,17]. At the same time, these products are characterised by an excellent safety profile. The most commonly reported adverse event in studies was abdominal pain, which occurred at a similar frequency as the placebo group [13,15,17].

Although BLs are increasingly considered adjunctive therapy in AR, their precise mechanism remains unclear. Because T helper (Th) lymphocytes play a central role in allergic disease immunopathogenesis, analysing their subpopulations may reveal the immunomodulatory effects of BLs in children with AR. Therefore, this study aimed to evaluate the impact of polyvalent mechanical BL (PMBL) therapy on the expression of Th cell-associated transcription factors and cytokines, reflecting Th1-, Th2-, Th10-, Th17-, and Treg-like immune responses, in children with grass pollen-induced AR.

## 2. Materials and Methods

### 2.1. Study Design

This study presents additional immunological analysis based on blood samples collected during a previously conducted randomised, double-blind, placebo-controlled trial (ClinicalTrials.gov ID: NCT04802616), designed to evaluate the efficacy of PMBL on the clinical course of AR and on the γδT, iNKT, and cytotoxic T cell subsets in children with grass pollen-induced AR. The parent trial was conducted in 2021 at three centres in eastern Poland and was approved by the Bioethics Committee of the Medical University of Lublin (Resolution No. KE-0254/251/2020). Written informed consent was obtained from the parents or legal guardians of all participants, and assent was obtained from the children, in accordance with the Declaration of Helsinki. More detailed information about the parent trial is available in reference [16]. The current study involved additional immunological analyses performed on blood samples collected during that trial. A research proposal for these investigations was submitted and subsequently approved by the same ethics committee (Resolution No. KE-0254/149/05/2022). The original informed consent covered the collection and future analysis of blood samples for immunological research purposes.

The objective of the present immunological analysis was to evaluate the effect of PMBL treatment on the expression of Th cell-associated transcription factors and cytokines related to Th1, Th2, Th10, Th17, and Treg-like immune responses in children with grass pollen-induced AR (seasonal AR [SAR]).

### 2.2. Participants and Intervention

The primary study enrolled children aged 5–17 years with a clinical diagnosis of SAR triggered by grass pollen, confirmed either by positive skin prick tests or elevated allergen-specific serum IgE levels. The participants were randomly assigned in a 1:1 ratio to receive either PMBL (Ismigen, Lallemand Pharma AG, Massagno, Switzerland) or a placebo. Each PMBL tablet contained 7 mg of BL derived from *Haemophilus influenzae*, *Klebsiella ozaenae*, *Klebsiella pneumoniae*, *Neisseria catarrhalis*, *Staphylococcus aureus*, *Streptococcus pneumoniae*, *Streptococcus pyogenes*, and *Streptococcus viridans*. The placebo was visually and organoleptically identical to the active product. The intervention was administered sublingually in three cycles, each consisting of 10 consecutive days of dosing followed by a 20-day drug-free interval. Detailed information on the inclusion and exclusion criteria, the randomisation and blinding procedures, and the sample size rationale has been described previously [16].

### 2.3. Immunological Analyses

Blood samples were collected at two scheduled visits during the 2021 clinical trial: visit 1 (V1), a screening and randomisation visit before the start of the grass pollen season, and visit 2 (V2), after completion of the study treatment and during the peak of the grass pollen season. Peripheral blood mononuclear cells (PBMCs) were isolated using density gradient centrifugation and stored at –80 °C until further immunological analyses.

**Figure 1:**
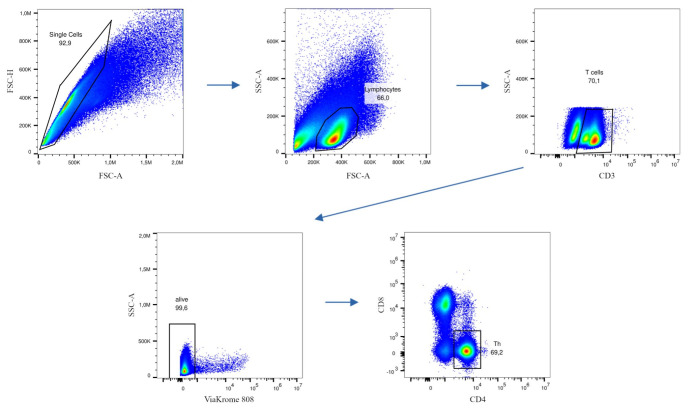
The gating strategy. First, singlets were gated based on FSC-A versus FSC-H; then, lymphocytes were selected among them. T lymphocytes were gated based on the expression of CD3; living cells were selected based on viability staining with ViaKrome 808. Finally, T helper (Th) lymphocytes were selected based on CD4 and CD8 expression.

Stored PBMCs were thawed and subsequently stained with a panel of surface antibodies listed in Supplementary Table S1, together with the viability dye Via Krome 808 (cat. no C36628, Beckman Coulter, Brea, CA, USA). After 20 min, the cells were fixed with paraformaldehyde and permeabilised with ice-cold methanol at −20 °C for at least 30 min. Then, the cells were washed twice with phosphate-buffered saline (PBS) with centrifugation for 10 min each time. Antibodies against intracellular targets were added, as listed in Supplementary Table S2. After incubation at room temperature for 30 min, the cells were washed with PBS and acquired on a CytoFlex LX flow cytometer (Beckman Coulter, Brea, CA, USA). Finally, the data were analysed with Kaluza v2.1.1 (Beckman Coulter, Brea, CA, USA). The gating strategy is presented in Figure 1.

### 2.4. Statistical Analysis

Statistical analyses were performed using SPSS Statistics version 25 (IBM Corp., Armonk, NY, USA). The chi-square test was used to assess whether the groups were comparable in terms of sample size. A two-way mixed-design analysis of variance (ANOVA) was conducted to evaluate differences between the two measurements within each treatment group and between corresponding measurements in the PMBL and placebo groups. Prior to the analysis, the assumptions of ANOVA were assessed. Normality of residuals was evaluated using the Shapiro–Wilk test, and homogeneity of variances between groups was assessed using Levene’s test. When significant interactions were detected, simple main effects analyses were performed with Bonferroni correction for multiple comparisons. A *p*-value < 0.05 was considered statistically significant.

## 3. Results

### 3.1. Participant Flow

The participant flow has been described in detail previously [16]. Blood samples from 41 children were available for this analysis (21 received PMBL and 20 received placebo). The mean age was 9.1 ± 2.6 years, 71% of participants were male, and most lived in urban areas (68%). All children were sensitised to grass pollen, with additional sensitisations including house dust mite (44%), pet dander (24%), weeds (10%), and moulds (10%). The groups were comparable at baseline.

### 3.2. Within-Group Changes in Transcription Factor and Cytokine Expression

Analysis of the expression levels of selected transcription factors and cytokines in Th lymphocytes, assessed based on the mean fluorescence intensity (MFI), revealed distinct within-group changes between V1 and V2.

**Figure 2:**
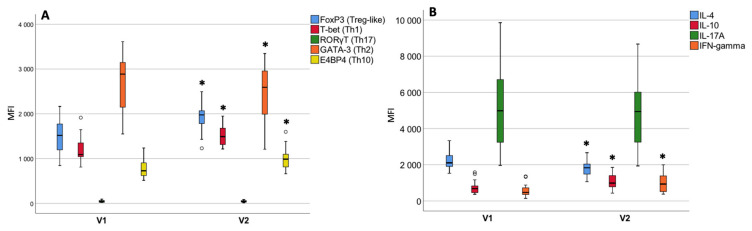
Changes in the expression of T helper (Th) cell-associated transcription factors (**A**) and cytokines (**B**) in the polyvalent mechanical bacterial lysate group. Data are presented as box plots of mean fluorescence intensity (MFI). The box represents the interquartile range, the horizontal line within the box indicates the median, whiskers represent the minimum and maximum values, and circles indicate outliers. Statistical analysis was performed using two-way mixed-design analysis of variance (ANOVA). An asterisk (*) placed above the V2 box plot indicates a statistically significant difference (*p* < 0.05) between V1 and V2 for the corresponding transcription factor or cytokine. V1, visit 1 (baseline); V2, visit 2 (after treatment).

In the PMBL group, there was a significant increase in the MFI for the transcription factors T-bet, E4BP4, and FoxP3 in Th lymphocytes at V2 compared with baseline (*p* < 0.001 for all). There was also a significant increase in the MFI for the cytokines IFN-γ and IL-10 (*p* < 0.001 for both). In contrast, there was a significant reduction in the MFI for GATA3 and IL-4 (*p* < 0.001 for both). Although RORγT and IL-17A expression decreased, these changes did not reach statistical significance. These findings are illustrated in Figure 2.

**Figure 3:**
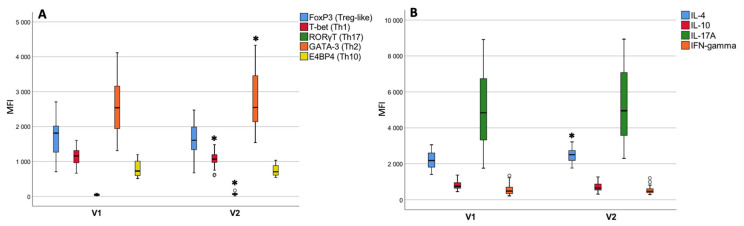
Changes in the expression of T helper (Th) cell-associated transcription factors (**A**) and cytokines (**B**) in the placebo group. Data are presented as box plots of mean fluorescence intensity (MFI). The box represents the interquartile range, the horizontal line within the box indicates the median, whiskers represent the minimum and maximum values, and circles indicate outliers. Statistical analysis was performed using two-way mixed-design analysis of variance (ANOVA). An asterisk (*) placed above the V2 box plot indicates a statistically significant difference (*p* < 0.05) between V1 and V2 for the corresponding transcription factor or cytokine. V1, visit 1 (baseline); V2, visit 2 (after treatment).

In the placebo group, there was a significant increase in the MFI for GATA3 (*p* < 0.001), RORγT (*p* < 0.001), and IL-4 (*p* = 0.004) between the visits. Conversely, there was a significant decrease in the MFI for T-bet (*p* = 0.007). E4BP4, FoxP3, IL-10, IL-17A, and IFN-γ did not show significant expression changes between V1 and V2. Figure 3 provides a visual representation of these findings.

### 3.3. Between-Group Differences in Transcription Factor and Cytokine Expression at V2

No statistically significant differences in the expression of the analyzed transcription factors or cytokines were observed between the PMBL and placebo groups at baseline (V1) (Table 1). At V2, there was significantly higher expression of the transcription factors T-bet (*p* < 0.001), E4BP4 (*p* < 0.001), and FoxP3 (*p* = 0.01) in Th lymphocytes from patients treated with PMBL compared with those receiving placebo. Similarly, the MFI for IFN-γ (*p* = 0.002) and IL-10 (*p* < 0.001) was significantly higher in the PMBL group. In contrast, there was significantly lower expression of RORγT (*p* = 0.01) and IL-4 (*p* < 0.001) in the PMBL group compared with the placebo group, while GATA3 and IL-17A expression were also lower but did not reach statistical significance. These differences, along with the statistical details, are reported in Table 1.

### 3.4. Association Between Immunological Parameters and Nasal Symptom Severity

**Figure 4:**
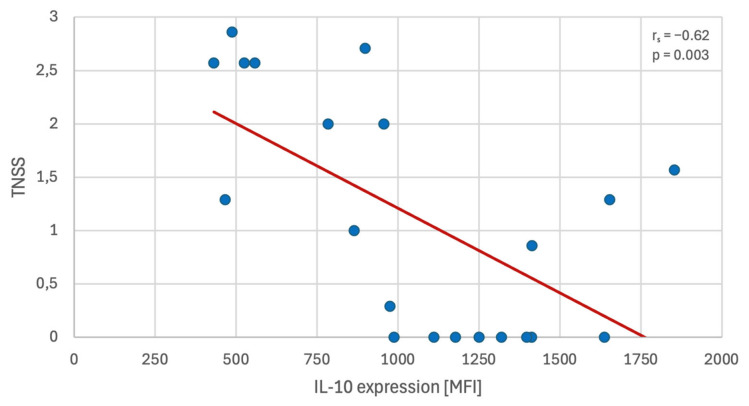
Correlation between post-treatment IL-10 expression in Th lymphocytes, expressed as mean fluorescence intensity (MFI), and the Total Nasal Symptom Score (TNSS) at the final follow-up assessment in the PMBL group. The association was analyzed using Spearman’s rank correlation test.

Additionally, the association between nasal symptom severity assessed using the Total Nasal Symptom Score (TNSS) and selected immunological parameters was evaluated in the PMBL group. Detailed clinical outcomes have been reported previously [16]. A significant negative correlation was found between post-treatment IL-10 expression in Th lymphocytes and post-treatment TNSS at the final follow-up assessment (Spearman’s rank correlation coefficient r_s_ = −0.62, *p* = 0.003), indicating that higher IL-10 expression levels after treatment were associated with lower symptom severity. This relationship is illustrated in Figure 4. No significant associations were observed between TNSS and the other analyzed cytokines.

## 4. Discussion

The present study evaluated the effects of PMBL therapy on the expression of Th cell-associated transcription factors and cytokines in children with grass pollen-induced AR. The results demonstrated that PMBL treatment increased the expression of transcription factors and cytokines associated with Th1-, Th10-, and Treg-like immune responses, while reducing those associated with Th2 responses. Furthermore, higher post-treatment IL-10 expression was associated with lower nasal symptom severity. Collectively, these findings support the proposed immunomodulatory effects of PMBL and are consistent with a shift from a Th2-dominant immune response toward a more regulated and potentially tolerogenic immune profile.

AR is characterised by a dysregulated balance between Th cell subsets, with a predominance of Th2-mediated pathways and increased production of cytokines such as IL-4, IL-5, and IL-13. These cytokines contribute to the hallmark features of allergic inflammation, including IgE production, eosinophilic infiltration, and enhanced mucus secretion [18,19]. In the present study, PMBL-treated patients showed significant upregulation of T-bet and IFN-γ, both indicative of Th1 polarisation. T-bet, a master regulator of Th1 differentiation, promotes IFN-γ expression while simultaneously repressing Th2 lineage commitment [20]. This pattern of expression suggests a reinforcement of Th1 responses following PMBL administration, which may contribute to counterbalancing the pathological Th2 dominance characteristic of AR.

This shift in balance was further reflected in the downregulation of the Th2-associated transcription factor and cytokine. In contrast to the upregulation of Th1-associated markers, we documented a significant reduction in GATA3 and IL-4 expression within the PMBL-treated group. This decline suggests suppression of Th2 responses, which, as previously noted, play a central role in the pathophysiology of AR [21,22]. The placebo group, by comparison, showed an opposite trend: an increase in Th2 and Th17 markers (GATA3, RORγT, and IL-4), along with a decrease in T-bet expression, suggesting progression of allergic inflammation during pollen exposure. Previous studies involving BLs in AR have suggested a shift from Th2- toward Th1-type responses as a potential mechanism underlying their clinical effects, based on observed changes in cytokine profiles such as increased IFN-γ and decreased IL-4 and IL-13 [11,12,13]. The present study extends these observations by demonstrating parallel changes in the expression of Th cell-associated transcription factors and cytokines.

In parallel, we observed increased expression of FoxP3, E4BP4, and IL-10 in the PMBL-treated group, consistent with enhanced activation of immunomodulatory pathways. FoxP3 is the defining transcription factor of classical Tregs, which suppress allergen-induced inflammation and help maintain immune tolerance [23]. IL-10 is a central immunoregulatory cytokine produced by multiple CD4^+^ T cell subsets, including both FoxP3^+^ Tregs and FoxP3^−^ IL-10-secreting cells. It limits immune activation by inhibiting antigen-presenting cell function, suppressing Th2 cytokine production, and reducing mast cell and eosinophil activity [24,25,26]. E4BP4 has been implicated in the regulation of IL-10 expression and may reflect the activation of IL-10-dependent regulatory circuits following PMBL administration. These findings suggest that PMBL may promote immune regulation in addition to modulating Th lymphocyte polarisation.

Between-group comparisons at V2 further support these observations. There were significantly higher levels of Th1-, Th10-, and Treg-associated markers (T-bet, E4BP4, FoxP3, IFN-γ, and IL-10) in the PMBL group, whereas markers of Th2 (IL-4) and Th17 (RORγT) were significantly lower compared with the placebo group. While the IL-17A levels did not differ significantly between the groups, the reduced expression of RORγT in the PMBL group may reflect a potential modulation of Th17 cell differentiation. Such changes in transcription factor expression could precede detectable alterations in cytokine production. This is of particular interest because the Th17 pathway has been shown to contribute to allergic inflammation by stimulating the release of Th2-type cytokines and facilitating the recruitment of eosinophils and neutrophils to mucosal tissues [27,28].

The relatively high variability observed in the expression of several transcription factors and cytokines most likely reflects the biological heterogeneity of the pediatric study population. Differences in immune maturation, individual immune responses and the degree of allergen exposure during the pollen season may all contribute to this inter-individual variability. These factors should be considered when interpreting the immunological findings.

Importantly, in the treatment group, higher post-treatment IL-10 expression was associated with lower nasal symptom severity at the final follow-up assessment. No such correlation was observed at earlier time points. This finding suggests that IL-10 may be related to the maintenance of clinical improvement over time and could be considered a potential marker of sustained treatment response.

In addition to modulating immunity, BLs have been suggested to support the restoration of the airway epithelial barrier, which is frequently compromised in allergic diseases [29]. This regenerative effect could be particularly relevant in the context of AR, where epithelial barrier disruption facilitates allergen penetration and amplifies downstream inflammation [22]. Although epithelial function was not assessed in the present study, the potential contribution of epithelial repair mechanisms cannot be excluded and may complement the immunological effects observed with PMBL treatment.

This study highlights the immunomodulatory potential of PMBL in children with SAR. The observed modulation of Th lymphocyte-related pathways suggests a shift in immune balance towards a more regulated and less allergy-prone phenotype. While some findings warrant further investigation, these results support the rationale for using PMBL as a complementary approach in allergy management, in line with the clinical benefits previously demonstrated in our studies [14,15,16].

Although this study provides valuable insight into PMBL-induced immune modulation in children with SAR, some limitations should be acknowledged. The sample size, while sufficient to detect significant immunological differences, limits the generalisability of the findings to broader age groups or sensitisation profiles. Moreover, the two-time-point design does not allow for assessment of the long-term trajectory of the observed changes. A further limitation of the present study is the absence of additional Treg markers, including CD25 and CD127, as well as memory differentiation markers such as CD45RA and CD45RO. Due to resource-related and technical constraints, the experimental design required the implementation of a single multicolor flow cytometry panel, necessitating prioritization of core markers. As a result, regulatory T cells were identified based on CD3, CD4, and FoxP3 expression. While FoxP3 is a defining transcription factor of the regulatory T-cell lineage, it can also be transiently expressed by activated Th cells. Consequently, the FoxP3^+^ CD4^+^ population described in this study should be interpreted as a Treg-like immunophenotype, rather than a functionally validated regulatory T-cell subset. The absence of functional suppression assays or stabilisation markers limits conclusions regarding the suppressive capacity and lineage stability of these cells.

## 5. Conclusions

PMBL administration in children with SAR significantly modulates the expression of Th cell-associated transcription factors and cytokines by enhancing the expression of Th1-, Th10-, and Treg-associated markers while reducing the expression of Th2-associated markers. These findings support the view that PMBL may exert immunomodulatory effects that shift the immune response towards a less allergy-prone and potentially more tolerogenic profile.

## Figures and Tables

**Table 1:** Expression of transcription factors and cytokines in T helper (Th) lymphocytes at baseline (V1) and after treatment (V2) in the PMBL and placebo groups.

Th-Associated Transcription Factor or Cytokine	PMBL (*n* = 21)		Placebo (*n* = 20)		Between-Group Comparison at V1 (*p*-Value)	Between-Group Comparison at V2 (*p*-Value)
	V1	V2	V1	V2		
T-bet (Th1)	1197.92 (279.49)	1500.66 (225.73)	1243.21 (461.18)	1055.08 (224.86)	0.70	<0.001
GATA3 (Th2)	2697.23 (626.65)	2475.87 (623.04)	2572.37 (841.26)	2731.48 (835.67)	0.59	0.27
E4BP4 (Th10)	768.25 (191.14)	988.32 (236.56)	805.82 (231.29)	743.91 (162.68)	0.57	<0.001
RORγT (Th17)	47.34 (24.82)	45.9 (22.17)	49.28 (24.38)	68.12 (32.29)	0.80	0.01
FoxP3 (Treg-like)	1501.29 (357.77)	1931.51 (321.4)	1664.82 (534.68)	1586.32 (488.97)	0.26	0.01
IL-4	2283.43 (495.09)	1781.17 (407.17)	2222.55 (471.62)	2491.87 (422.28)	0.69	<0.001
IL-10	741 (342.7)	1054.76 (422.91)	787.27 (224.76)	695.76 (248.24)	0.61	<0.001
IL-17A	5128.54 (2296.23)	4921.78 (2088.89)	5046.65 (2085.51)	5139.47 (2026.52)	0.91	0.74
IFN-γ	568.81 (327)	998.34 (485.31)	570.03 (313.58)	570.96 (293.26)	0.19	0.002

The data are presented as the mean fluorescence intensity with the standard deviation in parentheses. Statistical analysis was performed using two-way mixed-design analysis of variance (ANOVA). PMBL, polyvalent mechanical bacterial lysate; V1, visit 1; V2, visit 2.

## Data Availability

The raw data supporting the conclusions of this article will be made available by the authors on request.

## Supplementary Materials

The following supporting information can be downloaded at https://www.mdpi.com/article/10.3390/biomedicines14071623/s1. Table S1: Antibodies used for surface staining. Table S2: Antibodies used for intracellular staining.
