# Supplementary material for: Intervention with Polyvalent Bacterial Lysate Modulates T Helper Cell Subsets in Polish Children with Grass Pollen-Induced Allergic Rhinitis
Source: Biomedicines. 2026 Jul 19;14(7):1623. doi: 10.3390/biomedicines14071623 (PMC13406736; doi:10.3390/biomedicines14071623)
Supplement: Supplementary file 1 [file biomedicines-14-01623-s001.zip › biomedicines-4338469-supplementary.pdf]

Table S1. Antibodies used for surface staining.

**Target**	**Fluorochrome**	**Clone**	**Manufacturer**	**Catalogue number**
TCR V alpha 24 J alpha 18	FITC	6B11	BioLegend	342906
TCR V alpha 24 J alpha 18	PE-Cy7	6B11	BioLegend	342912
CD3	PE	UCHT1	BD Biosciences	555333
CD3	Krome Orange	UCHT1	Beckman Coulter	B00068
CD4	V450	RPA-T4	BD Biosciences	560345
CD8	Alexa Fluor 700	B9.11	Beckman Coulter	B76279
CD8	PE-Cy5	HIT8a	BioLegend	300909
TCR gamma/delta	PE-Cy5	IMMU510	Beckman Coulter	IM2662U
TCR gamma/delta	FITC	11F2	BD Biosciences	347903
CD45	BUV395	HI30	BD Biosciences	563792

Table S2. Antibodies used for intracellular staining.

**Target**	**Fluorochrome**	**Clone**	**Manufacturer**	**Catalogue number**
FoxP3	Pacific Blue	259D	Beckman Coulter	B90432
IFN-γ	PE-CF594	B27	BD Biosciences	562392
IL-10	BV786	JES3-9D7	BD Biosciences	564049
IL-17A	APC-Cy7	BL168	BioLegend	512320
IL-4	BV605	MP4-25D2	BioLegend	500828
E4BP4	PE	MABA223	Invitrogen	12-9812-42
T-bet	APC	4B10	BioLegend	644814
GATA3	PerCP-eFluor 710	TWAJ	Invitrogen	44-9966-42
RORγT	BV650	Q21-559	BD Biosciences	563424
